# The Biology of Chronic Pain and Its Implications for Pain Neuroscience Education: State of the Art

**DOI:** 10.3390/jcm12134199

**Published:** 2023-06-21

**Authors:** Kory Zimney, Wouter Van Bogaert, Adriaan Louw

**Affiliations:** 1 Department of Physical Therapy, University of South Dakota, 414 East Clark St., Vermillion, SD 57069, USA; 2 Pain in Motion Research Group (PAIN), Department of Physiotherapy, Human Physiology and Anatomy, Faculty of Physical Education and Physiotherapy, Vrije Universiteit Brussel, Laarbeeklaan 121, 1000 Brussels, Belgium; 3 Research Foundation–Flanders (FWO), Leuvenseweg 38, 1000 Brussels, Belgium; 4 Interuniversity Centre for Health Economics Research (I-CHER), Department of Public Health (GEWE), Faculty of Medicine and Pharmacy, Vrije Universiteit Brussel, Laarbeeklaan 103, 1000 Brussels, Belgium; 5 Department of Physical Medicine and Physiotherapy, University Hospital Brussels, Laarbeeklaan 101, 1000 Brussels, Belgium; 6 Evidence in Motion, 618 Broad Street, Suite B, Story City, IA 50248, USA

**Keywords:** chronic pain, pain neuroscience education, epigenetic factors, neural factors, endocrine factors, immune factors

## Abstract

Pain is an individualized experience for the person suffering from chronic pain. Significant strides have been made in the last few decades in understanding various biological changes that coincide with chronic pain. This state-of-the-art overview looks at the current evidence related to the biology of chronic pain and the implications these findings have on the delivery of pain neuroscience education (PNE). The paper summarizes the various (epi)genetic, neural, endocrine, and immune factors discovered and explored in the scientific literature concerning chronic pain. Each of these biological factors has various implications for the content and delivery of PNE. We discuss the future directions these biological factors have for the clinical implementation of PNE by linking the importance of behavior change, optimizing the learning environment, and using an individualized multimodal treatment approach with PNE. In addition, future directions for research of PNE based on these biological factors are provided with importance placed on individualized patient-centered care and how PNE can be used with traditional modes of care and growing trends with other care methods. PNE was originally and continues to be rooted in understanding chronic pain biology and how that understanding can improve patient care and outcomes.

## 1. Introduction

Thomas Kuhn’s The Structure of Scientific Revolutions [1] pointed out two primary mechanisms required for science to advance. One is the gradual accumulation of knowledge or facts; the other is the rapid shift in integrating the facts that occurs when a new theory or paradigm is proposed. In the scientific area of the biology of chronic pain, continual advancements in understanding the basic facts of the biology within patients suffering from chronic pain have occurred through the decades. One such advancement led to the initial paradigm shift in patient education by Louie Gifford and David Butler [2] with the concept of educating patients about pain, not just their injury, during clinical practice. Later, the idea of explaining pain was formally introduced into the research literature through a randomized control trial by Lorimer Moseley [3]. Following this first trial, the paradigm shift led to an explosion of research and facts around the benefits of pain neuroscience education (PNE) in the past two decades.

PNE can be described as different educational methods used with individuals to change someone’s understanding of pain. It uses various change strategies, psychologically informed practices, and modern pain-related biological science to elicit conceptual change within the individual to reduce fear, anxiety, and worry about their pain condition. This shift in the conceptual understanding of pain for an individual can then lead to alterations in their attitudes, beliefs, and behaviors [4].

To date, numerous systematic reviews and meta-analyses have shown the benefits of PNE in various areas, such as self-reported pain reduction, lower disability, decreased fear-avoidance and pain catastrophizing, improved pain knowledge, increased movement, and lower healthcare costs [5,6,7,8,9,10,11,12,13]. While there is evidence in place showing that PNE has positive benefits, there are still other studies that show little to no effects with the use of PNE [14,15]. Future work needs to continue exploring nuances of education and therapist-patient interaction during the educational process to improve the outcomes with the use of PNE and when it may provide benefit and when it will be less useful. Research shows small to moderate effect sizes with the general use of PNE when delivered within a multimodal treatment plan, typically combined with exercise. Unfortunately, the exact dosage regarding the amount of information, length of time to deliver the education, and the best setting, whether in groups or individual, is still unknown. To improve these effect sizes, the individualization of PNE may need to be tailored to the individual patient in front of us and their specific biological, emotional, and social needs. Educational strategies, such as PNE, also need to be delivered with care as potential nocebo effects can occur [16,17]. Pain is an individual human experience; thus, the care for an individual needs to be on a personal level [18]. This is sometimes at odds with much of current healthcare practice and payment systems that want to reduce treatment delivery into simpler, linear models and methods, specifically since these can be more easily controlled for direct cause and effect research purposes, standardized for ease of delivery, and monitored more closely for payment. One thing that the study of the biology of pain has taught us is that simple does not fit into the model or method of treatment very well, but complex and nonlinear models and methods of treatment do [19,20].

This state-of-the-art paper provides an overview of the current evidence regarding the biology of chronic pain and the implications for PNE for people with chronic pain within the new paradigm shift of understanding and educating individuals on the complexity of pain. Although the biology of chronic pain literature is extensive, this paper aims to highlight a few of the significant biological discoveries in the past few decades and how they continue to shape the delivery of PNE. The primary areas covered will be genetic (more specifically epigenetic), neural (primarily neuroplasticity and processing within the brain), endocrine (related to autonomic responses to stress and sleep), and immune factors. While there are other factors potentially involved for people with chronic pain, this paper will explore specifically these four factors in terms of how they pertain to pain education content and delivery changes within this new paradigm. 

## 2. State of the Art

Pain is an unpleasant sensory and emotional experience associated with, or resembling that associated with, actual or potential tissue damage [21]. In the revised 2020 International Association for the Study of Pain definition, key notes were added. One of those solidified the idea that pain is a complex process influenced by varying biological, psychological, and social factors. This state of the art will further explore this complicated intertwining of these regarding how psychological and social factors can change biology and how biological factors can affect psychology and social aspects (Table 1).

### 2.1. (Epi)genetic Factors

Genetics plays a role in pain, especially in those with chronic pain, as genetic risk factors have been found in several chronic pain conditions [22]. The various genes associated with chronic pain are long and complex, including genes from serotonergic, glutamatergic, GABAergic, cytokines, growth factors, and more [23,24,25]. Though genetics is an essential factor in someone’s pain experience, it alone cannot explain the whole picture, as demonstrated through multiple twin studies [26,27]. Another scientific finding currently at the center of modern medicine is epigenetics [28]. Epigenetics has shown us that gene expression is not solely based on someone’s genetic background. Instead, the environment and the individual’s health also influence genetic expression. A common metaphor that can be used to explain the relevance of epigenetics is to consider people’s genetic structure as a full set of piano keys, with epigenetics being the mechanism determining which keys are being played [29]. This understanding requires us to take a much broader look into someone’s health and pain condition and look beyond the body, considering their lived environment and contextual factors [30,31,32,33]. Indeed, current evidence shows that physical activity and psychological stress (e.g., fear) can induce epigenetic changes in relation to pain [29]. Whereas physical activity was found to positively influence the (epi)genetic processes regarding nociceptive modulation, stress response, and the pathophysiology of chronic diseases, intense psychological stress seems to negatively influence such processes, which can even result in increased pain sensitivity [29,34]. Moreover, such stress-induced changes seem to be maintained long after the stressful event has ended [34]. Additionally, epigenetics is also suggested to play a role in the transition from acute to chronic pain, as well as the neuroplasticity responsible for the hyperexcitability of the central nervous system, which is often present in people with chronic pain [34,35,36]. The importance of such knowledge on epigenetics in PNE is to help patients understand the intricacies of genetics that might make them more sensitive to pain and how the environment can change the expression of those genes through the epigenetic process. Specifically, clinicians can emphasize the role of environmental and lifestyle factors in the complexity of pain sensitivity. Clinicians can also assist patients in understanding that some individuals might be more genetically prone to being hypersensitive and that epigenetic influences can amplify or limit this predisposition. Overall, the care for people with chronic pain needs to extend to the larger conversation regarding social determinants of health and its role in an individual’s experience but also the larger societal issues of their role in the pain epidemic [30,31,32]. This provides a ripe ground for a powerful combination of acceptance and understanding of their biological response to the condition yet hope for change within other factors they control. 

### 2.2. Neural Factors

Looking at neural-related changes during pain led to the introduction of Gate Theory, one of the most significant paradigm shifts in the study of pain [37]. The idea that neural processing could be altered and changed at different levels was novel then. This idea regarding neural processing changes at the spinal cord level has been a springboard into the complexity of the pain experience. This theory pointed out that the simple cause-and-effect process does not occur with pain, especially as pain persists. Once this shift occurred to recognize that pain was not a cause-and-effect mechanism, it opened study into neuroplasticity and memory at multiple levels, from the peripheral receptor at various points through the nervous system all the way up to the brain. It is well understood that the biology of pain changes the structure and function of the nervous system, which also changes the pain experience [38,39,40,41]. Hasmi, et al. [40], showed a dramatic shift in information processing as pain persists and that emotional circuits become much more activated over time during the pain experience. Other research has also shown us that pain changes the brain, and other social determinants of health can also affect brain development [42,43]. These two findings have had profound implications on PNE. As pain persists, we need to consider the emotional needs of our patients, and education should be directed in that area. In addition, the social determinants of an individual have a significant effect on their health. Thus, attention must be placed in that direction during our educational and treatment process. This emphasis also ties into the need to consider the patient’s emotional state when delivering PNE and potential interactions when utilizing mindfulness stress-based reduction techniques in conjunction with or before education [44].

Another discovery area specific to neuroplasticity and chronic pain is the functional and structural changes to the spinal and cortical representation of the patient’s body image and schema, along with tactile acuity abilities [45,46,47,48,49,50,51,52]. These findings highlight the importance of evaluating tactile acuity [46] and motor imagery [53] with patients suffering from chronic pain along with the value of utilizing graded motor imagery techniques and sensory discrimination training [51,54,55,56]. Explaining these concepts to patients is integral to the treatment process. Compliance with self-management is vital with these interventions because of the repetition needed for beneficial neuroplastic changes. Patient education and support have been linked to improved compliance with chronic conditions [57]. Helping the patient understand these conscious and unconscious representation alterations of their body can provide an essential link in their understanding of their “abnormal” feelings and awareness of the affected area of the body as “normal” consequences of neurobiological changes that can occur in the body especially as pain persists. This deeper understanding can be a motivational catalyst for carrying out self-management with the interventions.

Another component of the “dark side” of neuroplasticity as it relates to chronic pain is involved with memory and learning [58]. These neuroplastic changes involved with pain memories deepen the argument that chronic pain is a disease of the nervous system and which distinguishes itself from the phenomena of acute pain and notification of tissue injury. The idea of pain memories has increased in acceptance since the early experiments within Melzack’s lab at McGill University [59]. Recent research has shown that pain threshold levels [60] and muscle strength [61] are altered in individuals after injury compared to those without a history of injury. This finding has implications within PNE as part of the educational process to help patients understand how the performance of activities will involve overcoming these painful memories and retraining the nervous system.

### 2.3. Endocrine Factors

The relationship between stress and pain has long been established, with stress being able to induce either hyper- or hypoalgesia as well as allodynia in patients [62,63]. The concept of increased nerve sensitivity (hyperalgesia and allodynia) is a clinical presentation that does not imply a mechanism but has been identified during studies where individuals have reduced pain pressure thresholds when encountering a stress enhanced environment or situation [64,65]. The analgesic effect of stress depends on the type of stressor, but it can also differ between patients, particularly those with chronic pain [66]. Besides pain modulation, stress can induce or worsen other complaints, such as fatigue or cognitive symptoms, in people with chronic pain [67,68,69]. Such induction or worsening of symptoms due to stress can be described as stress intolerance [68]. Recently, Wyns, et al. [68], have provided an excellent overview of how stress intolerance plays a significant role in chronic pain. Two of the primary hormonal outputs of the endocrine system during the Hypothalamus–Pituitary–Adrenal (HPA) activation are cortisol and adrenaline. The paradigm shift from the pain education level with this knowledge is the importance of reducing stress and improving the environment to allow learning to occur [62]. The increased threat of pain enhances increased sensitization with associative fear learning, and it can amplify pain [70]. This concept that stress and emotions play a significant role in pain experiences must be a significant component of the educational process [71]. Not only is it a relevant concept to discuss with the patients as part of their education, but it can also aid clinicians in establishing a proper context to provide the education. Creating a safe learning environment is vital for the patient to learn the ideas of pain science and understand that it is more about the nerve sensitivity than the state of the tissues [72]. Another valuable concept in PNE is creating an optimal learning environment. Alterations of endocrine function can influence the learning environment. Being in pain can impair a patient’s value-based goal-directed behavior [73] through the effects of the stress response system. This response can be mediated by the latter’s influence on the prefrontal cortex neural networks [74]. As such, PNE needs to be directed toward not just the accumulation of knowledge about pain by the patient but relevant knowledge that will spark goal-directed behavior to improve their functional status. 

The endocrine system is also closely linked to sleep, as it is influenced heavily by circadian rhythms and sleep–wake states that alter hormonal control within individuals [75,76]. These alterations in endocrine function associated with sleep disturbances and chronic pain are connected to the Hypothalamus–Pituitary–Adrenal (HPA) axis which mediates an individual’s response to both physical and psychological stress [77]. The alterations in the HPA axis have been found to affect cortisol levels leading to various pain sensitivity problems [78]. The endocrine changes in cortisol levels due to sleep disturbance and chronic pain are also tightly interrelated with the immune system and change with pro-inflammatory cytokine production. Another interesting link between chronic pain and sleep disturbance is melatonin production. Some individuals suffering from various chronic pain conditions have seen improvements in pain when taking exogenous melatonin [79,80]. This understanding of the links between chronic pain and sleep disturbance can be vital while delivering PNE to help patients make meaningful connections to various health changes related to endocrine function changes and provide reasoning behind the importance of sleep hygiene within a complete multimodal treatment plan.

**Table 1:** Evidence regarding biological factors involved in pain and implications for pain neuroscience education.

Biological Factor	First Author, Reference	Investigated Mechanism	Implication for Pain Neuroscience Education
(Epi)genetic	Zorina-Lichtenwalter [17]	Genetic contributions to chronic pain	Educate the patient on the role of genetic factors in the variability of stimuli responses.
	Polli [24]	Physical and psychological stressors can induce epigenetic changes in relation to chronic pain	Explain to the patient how various stressors can alter genetic expression to explain why certain stimuli can be experienced differently in other contexts.
	Nirvanie-Persaud [29]	Epigenetics play a role in the transition from acute to chronic pain	Provide understanding to the patient of how various factors, including genetic and environmental, may have led to persistent pain.
	Mauceri [30]	Epigenetic changes can facilitate peripheral and central sensitization processes	Explain to the patient how their increased sensitivity can partially be explained and maintained by changes on the genetic level.
Neural	Hasmi [35]	Shift of pain processing from nociceptive to emotional circuits with chronification of pain	Consider and discuss emotional components during the patient’s education and care, especially as pain persists.
	Bosnar Puretic [36]	Neuroplasticity can lead to central sensitization process	Educate the patient on the key concept of neuroplasticity and how the nervous system changes and sensitizes over time to help focus more on the sensitivity of the nervous system and less on damage to the tissues as pain persists.
	Catley [41]	Spinal and cortical representation changes in people with chronic pain	Educate the patient on the concept of body representation changes that can occur with chronic pain and the need for various interventions (i.e., GMI and sensory discrimination) to facilitate recovery.
	Price [53]	Linking of pain and memory mechanisms with chronic pain	Provide understanding to the patient of the concept of “pain memories” and how treatment needs to work on overcoming pain memories that might be maladaptive to function.
Endocrine	Lunde [58]	Stress response system implications within chronic pain	Educate the patient on the link between chronic pain and the stress response system to provide an understanding of the individual’s pain experience.
	Wyns [61]	Stress intolerance role in chronic pain	Explain to the patient why various stress management interventions can assist in improving chronic pain limitations.
	Haack [70]	Sleep deficiency and chronic pain alterations in endocrine function	Educate the patient on the important link between poor sleep and changes in endocrine function.
Immune	Marchand [74]	Inflammatory mediators released from immune cells contribute to persistent pain states	Educate the patient on the link between the immune system and chronic pain is critical. These facts also help explain why pain may increase or decrease based on immune system response and may not be due to tissue changes.
	Totsch [76]	Diet can influence pain through the immune system	Include education on why diet changes in a multimodal treatment could be beneficial.
	Besedovsky [77]	Sleep can influence pain through the immune system	Educate the patient on improving sleep hygiene as part of the multimodal treatment.

### 2.4. Immune Factors

The immune system has a very prominent role in chronic pain [81,82]. Research has found a long list of inflammatory molecules involved in the experience of pain (e.g., mast cells, cytokines, macrophages, neutrophils, and T and B cells). In addition, extensive study has investigated various immune mediators and cytokines that can alter pain processing (e.g., TNFα, IL-1β, NGF, bradykinin, serotonin, and chemokines). Understanding an individual’s complex immune system processes opens an extensive door as part of the educational process with the patient. Appreciating this complexity helps them understand further that their body is not damaged but is overprotective and can be retrained [71]. In addition, understanding the immune system’s involvement in chronic pain has also been linked to the importance of dietary interventions to reduce the potential inflammation-mediated disorder [83]. Not only diet, but sleep [84] and stress reduction through touch [85] and meditation [86,87,88] also have links to immune system function and pain. These findings further support using a multimodal approach with PNE to maximize the effects of any one treatment through the combined impact of linking treatments to improve the individual’s health on multiple levels. It also provides an explanation of why previous individual treatments had little to no effect but still might be beneficial. A common metaphor to explain this is that a car with four flat tires does not run well unless all four tires are inflated properly. Pumping up one tire is an important step in the process, but until the other three tires are inflated, it will appear as if the efforts made pumping up one tire were meaningless. This is true for important interventions: exercise, sleep hygiene, stress reduction, diet, etc., alone may seem pointless but when all of them are working, positive changes can be seen.

## 3. Future Directions for Clinical Practice

**Figure 1:**
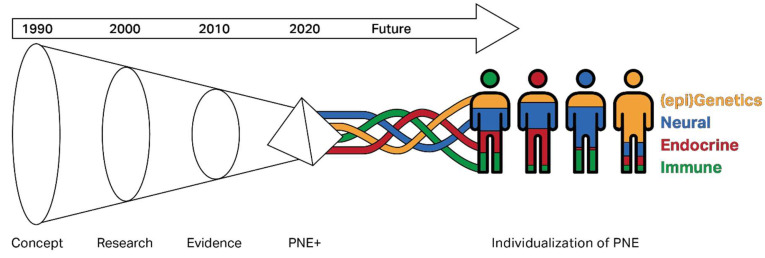
Timeline of PNE in clinical practice with future directions.

Understanding the complex biological systems interaction that interplays in chronic pain has carried over to changes in treatment, especially around PNE. Figure 1 depicts the timeline of PNE from the 1990s with a gradual refining of the use of PNE over the decades. PNE has continued to evolve from its early days as a clinical concept in the 1990s, moving into the initial research testing phase in the 2000s, and becoming more widely accepted through the depth of evidence supporting the use of PNE from 2010 to 2020. Future directions will need to look at PNE plus the other multimodal treatments in conjunction with each other within a patient-centered approach to care. During this more individualized approach, PNE plus the other interventions must tie together the biological factors (epigenetic, neural, endocrine, immune, and others) that work at different levels in each patient encountered during clinical practice. The various colors within the figure symbolize the potential different levels at which each of these factors may be involved with individual patients (Figure 1).

PNE is not about the result of making the patient more knowledgeable about pain; more importantly, it is about the process of using the knowledge gained to facilitate behavior change in a complex environment [71,89,90,91,92]. Part of the behavior change for patients is the reconceptualization process of their pain experience through a deeper understanding of the complex biology occurring within themselves [71,72,90] Some excellent qualitative studies looking into the reconceptualization process can be constructive for clinical practice. They recognized that patients would go through various degrees of reconceptualization [90]. Patients go through their journey to reconceptualize their pain experience from one bound in a biomedical viewpoint toward a broader biopsychosocial view. Within the roots of the reconceptualization process is the psycho–neuro–endocrine–immune changes occurring within the patient’s biology. Since the patient needs to undergo this change process, the clinician must understand the stages of change that patients will go through because the educational needs at each step differ [93,94,95]. Clinicians need to consider the various processes of change and utilize skills and techniques to help patients progress in the change process (e.g., consciousness-raising, self-reevaluation, counterconditioning, helping relationships, and self-liberation) [95]. Another important factor is that patients must find personal relevance in their education [90]. Pain knowledge alone is useless to patients unless they find meaning specific to their condition. Stories and metaphors are a mainstay in PNE, but these stories must make sense to the patient in their context, not the clinicians [96,97]. Skillful patient history-taking is needed to explore the patient’s prior level of beliefs about their condition and the treatments that might be beneficial [90,98]. For some patients, it will be essential to dispel previous myths (de-educate) before moving forward with new knowledge (re-educate) and helping them reconceptualize their pain experience [99].

Keeping in mind that the change in the various biological factors discussed happens concurrently as part of the behavior change process during PNE, integrating motivational interviewing is a skill clinicians should consider using to assist with this behavior change process. When motivational interviewing techniques are implemented, the needed biological changes can occur [100]. The reader should review the manuscript by Nijs, et al. [91] for a complete practical guide for clinicians along with Miller and Rollnick’s book: Motivational Interviewing: Helping people change [101]. Motivational interviewing is a crucial behavioral strategy used to assist patients in the behavior change process and sets the stage for the patient to be more receptive to many of the concepts of PNE to assist in the pain reconceptualization process [102]. The general qualities of motivational interviewing are essential to carry through the pain education process [101]. Motivational interviewing and PNE should contain a guiding style of communication that fits between good listening and giving information. PNE and motivational interviewing should also be designed to empower the patient to change by drawing out their own meaning and capacity for change. Lastly, both should be based on a respectful and curious way of being with people that facilitates the change process and honors the patient’s autonomy. The motivational interviewing fundamental methods of engaging, focusing, evoking, and planning can help the clinician create the “flow” of conversation through the educational session. 

For behavior change to occur, the clinician needs to assist in creating the right environment for the patient. The right environment encompasses physical, emotional, learning space, and psychologically safe aspects. Patients with chronic pain need to be open to change; they need safety, and their physical and, potentially more importantly, emotional needs must be met [92]. Research has shown that meeting the patient’s emotional needs is often the most critical as the relationship between the provider and the patient progresses along with the length of time the patient has their condition [103]. The clinician needs to see the biological links and importance of the neural, endocrine, and immune systems in the concept of the right environment playing a role in the treatment process. The famous quote of Theodore Roosevelt that addresses this concept is fitting to remember: “No one cares how much you know, until they know how much you care”. Meeting these emotional needs of the patient is grounded in our understanding of the basic science of the shift in brain processing as the pain becomes chronic in the more emotionally related brain areas [42]. The emotional safety of an individual is built through the reciprocal nature of trust within the healthcare relationship [104,105]. Trust is a vital component of the therapeutic alliance [106,107]. PNE that provides an evidence-based biological understanding of the patient’s pain experience, that answers the patient’s questions, and that helps them make sense of their pain can be a vehicle to build trust between the patient and provider [97,108].

**Figure 2:**
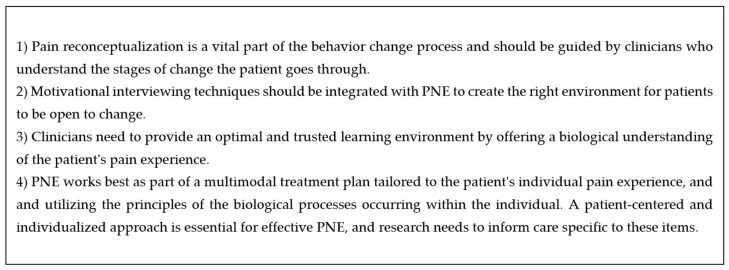
Key messages for clinical practice.

PNE alone for a complex dynamic systems problem such as chronic pain has little effect when delivered in isolation [6]. The evidence continues to support that PNE works best when delivered as part of a larger treatment plan tying together all the treatment options (nutrition, sleep, stress reduction, meditation, breathing, exercise, manual therapies, etc.) into a coherent message providing hope. All these treatment interventions can work in a symbiotic nature when we recognize the principles of the biological processes occurring as we deliver each mode of treatment. When we embrace a complexity mindset to chronic pain resulting from complex, dynamic, and individually unique interactions between the various factors within the more extensive system [20], it allows for a way toward better health and recovery from chronic pain, i.e., when we can utilize a variety of treatments that have various interactions within an individual and their biology and overall pain experience. Although, clinically, therapists have to appreciate that there is heterogeneity in each individual’s pain problem, there are also overlapping items that can help us classify pain mechanisms [109,110,111,112,113,114] and lead the clinician toward various treatment options that are the most plausible for improved outcomes (Figure 2).

## 4. Future Directions for Research

The next evolution in PNE research needs to move beyond the general question of whether it works. If we fully embrace the multidimensional nature of chronic pain, we need to recognize the inherent limitation of any research directed toward a single domain and expect meaningful outcomes. A one-size-fits-all approach is most likely not going to suit any specific individual. The NIH National Pain Strategy points to the need for care to be patient-centered, compassionate, and individualized for every patient. If this is the treatment goal, PNE research needs to inform care specific to these items. 

PNE must break from the idea of rote intervention employed equally to all patients. Future studies should explore the uniqueness and complexities of each patient and how this affects the communication style of the therapist, the patient-centered content to be delivered, and the individualized value-centered goals and outcomes of the education. Specific studies are needed to examine how the patient’s various personal and social factors and the concordance of those factors with the therapist can alter the outcomes and the potential content that needs to be provided along with the delivery style. More patient-centered research is necessary to examine learning styles, stages of behavior change, levels of therapeutic alliance, implicit biases, and other factors that can alter the learning experience and how that will affect the outcomes for the patient receiving the interventions. Because of the various interactions with other treatments, ongoing research needs to explore the effects of diverse PNE approaches and interactions when provided with multiple treatment interventions. Given the ongoing digitalization of healthcare, studies examining the possibilities of eHealth for PNE are needed. Specifically, the cost-effectiveness of such eHealth applications to deliver an individually tailored education to (multiple) patients would be worthwhile to investigate. In addition, the study designs will require more pragmatic approaches of using multiple methods to develop these complex interactions [115,116]. These designs will require nonlinear and iterative development of interventions. Different methodologies should be considered, such as cluster randomized trials, stepped-wedge designs, or preference trials. 

**Figure 3:**
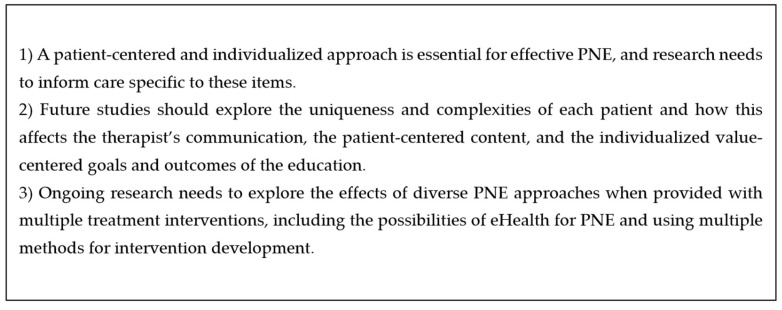
Key messages for research.

Because of the individualized experience of each patient, ongoing phenomenological and grounded theory qualitative study needs to be conducted to assist the clinician and the researcher in embracing and fully understanding the patient’s pain experience. This enhanced understanding can lead to a greater non-judgmental and empathetic understanding of the patient, thus enhancing the therapeutic alliance [117] (Figure 3).

## 5. Conclusions

This state-of-the-art paper has explored some of the more relevant advances in the biological understanding of pain related to (epi)genetic, neural, endocrine, and immune factors. The depth and breadth of our knowledge of these factors have grown substantially in the past few decades. This evidence has and should be used to help patients understand the complexity of pain, especially as it persists. The use of PNE is rooted in assisting patients in understanding these complex biological processes occurring in them by utilizing stories and metaphors to help them move through a behavior change process to improve function and potentially reduce pain as they regain quality of life.

## Data Availability

Not applicable.
